# Changes in skeletal and dental relationship in Class II Division I malocclusion after rapid maxillary expansion: a prospective study

**DOI:** 10.1590/2176-9451.19.3.075-081.oar

**Published:** 2014

**Authors:** Carolina Baratieri, Matheus Alves Jr, Ana Maria Bolognese, Matilde C. G. Nojima, Lincoln I. Nojima

**Affiliations:** 1 Professor, Department of Orthodontics, Federal University of de Santa Catarina, UFSC.; 2 PhD resident in Orthodontics, Federal University of Rio de Janeiro.; 3 Full professor, Department of Orthodontics, Federal University of Rio de Janeiro.; 4 Professor, Department of Orthodontics, Federal University of Rio de Janeiro, UFRJ.

**Keywords:** Palatal expansion technique, Angle Class II malocclusion, Clinical trial, Orthodontics

## Abstract

**Objective:**

To assess skeletal and dental changes immediately after rapid maxillary expansion (RME) in Class II Division 1 malocclusion patients and after a retention period, using cone beam computed tomography (CBCT) imaging.

**Methods:**

Seventeen children with Class II, Division 1 malocclusion and maxillary skeletal transverse deficiency underwent RME following the Haas protocol. CBCT were taken before treatment (T_1_), at the end of the active expansion phase (T_2_) and after a retention period of 6 months (T_3_). The scanned images were measured anteroposteriorly (SNA, SNB, ANB, overjet and MR) and vertically (N-ANS, ANS-Me, N-Me and overbite).

**Results:**

Significant differences were identified immediately after RME as the maxilla moved forward, the mandible moved downward, overjet increased and overbite decreased. During the retention period, the maxilla relapsed backwards and the mandible was displaced forward, leaving patients with an overall increase in anterior facial height.

**Conclusion:**

RME treatment allowed more anterior than inferior positioning of the mandible during the retention period, thus significantly improving Class II dental relationship in 75% of the patients evaluated.

## Abstract (pt)

**Objetivo:**

avaliar, por meio de imagens de tomografia computadorizada de feixe cônico (TCFC), as mudanças esqueléticas e dentárias, imediatas e após 6 meses de contenção, causadas pela expansão rápida da maxila (ERM) em pacientes com má oclusão de Classe II, divisão 1.

**Métodos:**

dezessete crianças com má oclusão de Classe II, divisão 1, e deficiência transversal da maxila, foram submetidas a ERM, de acordo com o protocolo proposto por Haas. TCFC foram realizadas antes da ERM (T_1_), imediatamente após a fase ativa (T_2_) e após 6 meses de contenção (T_3_). Alterações anteroposteriores (SNA, SNB, ANB, *overjet* e RM) e verticais (N-ANS, ANS-Me, N-Me e *overbite*) foram analisadas.

**Resultados:**

imediatamente após a ERM, enquanto a maxila se deslocou para frente, a mandíbula se movimentou para frente e para baixo, aumentando o *overjet* e diminuindo o *overbite*. Durante o período de contenção, a maxila retornou para posterior e a mandíbula deslocou em direção anterior, aumentando a altura facial anterior.

**Conclusão:**

a realização da ERM permitiu que a mandíbula se posicionasse mais anteriormente do que inferior durante o período de contenção, melhorando a relação molar de Classe II em 75% dos pacientes avaliados.

## INTRODUCTION

Angle^1^ defined Class II malocclusion as the distal relationship of the lower first molar in relation to the upper first molar. Studies have recently shown that in addition to the anteroposterior and vertical problems related to Class II malocclusions, posterior transverse discrepancy is also frequently associated with it.^2^

Diagnosis of posterior transverse discrepancy often passes unnoticed at clinical examination as this problem is camouflaged by the Class II skeletal pattern. The characteristics of Class II malocclusion, in all three spatial planes, pre-exist in deciduous dentition and persist into mixed dentition without correction.^3^ As soon as transverse maxillary deficiency is diagnosed, rapid maxillary expansion (RME) should be implemented regardless of other skeletal alterations because transverse maxillary growth ends earlier than growth in other directions.^4^

The majority of studies assessing RME outcomes showed that the mandible rotated downward and backward,^5^ which is usually an unwanted effect in Class II patients. Clinical observations and case reports reveal either an improvement or correction of the sagittal relationship in Class II patients during the retention period following RME.^6^

Cone beam computed tomography (CBCT) allows a complete scan of the face within a few seconds, with less ionizing irradiation than CT^7^ or full-mouth radiographic survey for orthodontic diagnosis.^8^ Recent technological advances in dental software allow cephalometric concepts and tools to be combined with CBCT advantages.

Despite a large number of studies reporting on the effects of RME, most of them failed to specify or distinguish the type of malocclusion (Class I, II or III) in the subjects evaluated. Accordingly, there is a lack of information surrounding Class II malocclusion patients who underwent RME as the only treatment intervention. Therefore, the aim of this study is to use CBCT imaging to assess changes in dental and skeletal relationships in Class II, Division 1 malocclusion patients immediately after RME as well as after a 6-month retention period.

## MATERIAL AND METHODS

This prospective study was carried out in the Department of Orthodontics of the Federal University of Rio de Janeiro with the approval of the Institute of Collective Health Studies Research Ethics Committee (ref.128/2009-0052.0.239.000-09) and with an informed consent form signed by patients and parents.

Seventeen white Brazilian subjects (8 boys and 9 girls with mean age of 10.67 and 10.05 years old, respectively) presenting Class II Division 1 malocclusion and maxillary transverse skeletal deficiency were selected and diagnosed to receive RME therapy. In addition, patients were followed for the following six months.

In selecting the sample, the following inclusion criteria were applied: Chronological age ranging from 7 to 12 years old; overjet greater than 3 mm; Class II molar (unilateral or bilateral) and skeletal (ANB ≥ 4º) relationship; maxillary skeletal transverse deficiency (distance from J point to facial frontal line > 12 mm);^9^ skeletal maturation CS1 through CS3 as evaluated by the Cervical Vertebral Maturation method.

All patients were submitted to RME following the Haas protocol.^4^ The appliances were standardized with stainless steel wire, 0.047-in in diameter (Rocky Mountain Orthodontics) and expansion screw of 11 mm (Dentaurum, Magnum - 600.303.30). Upon insertion, the expansion screw was activated four turns (0.2 mm per turn) on the first day, and on the following days it was activated two turns per day, (0.4 mm daily). The active phase varied from 2 to 3 weeks, depending on the individual maxillary transverse deficiency originally diagnosed. Afterwards, the expander screw was stabilized with a 0.012-in double thread ligature and was passively kept in place for the following six months after which the appliance was removed.

CBTC scans were taken before treatment (T_1_), immediately after stabilization of the expansion screw (T_2_), and after removal of the expander (T3). The scans were performed with the same cone beam machine (i-CAT, Imaging Sciences International, Hatfield, Pennsylvania, USA), according to a standard protocol (120 KVp, 3 mA, FOV 13x17 cm and voxel 0.4 mm). Volume data at T_1_, T_2_, and T_3_ were exported in DICOM (digital imaging and communication in medicine) format into Dolphin Imaging software^®^ (Charsworth, Calif, USA).

**Figure 1:**
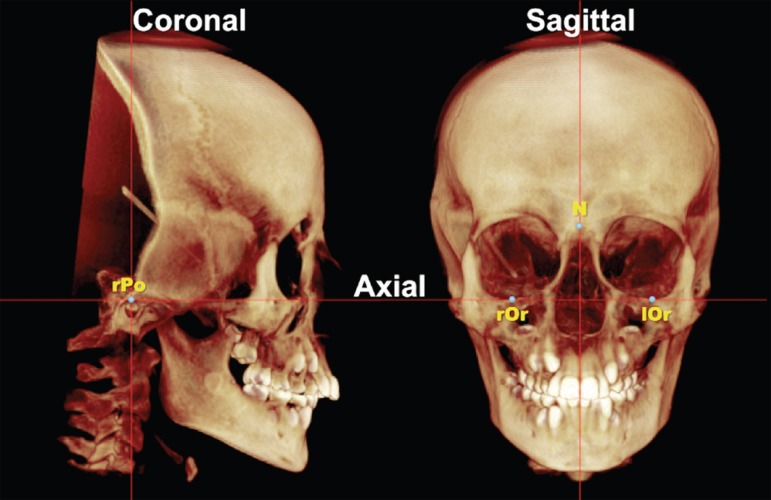
3D digital image of the head after orientation by axial, coronal and sagittal planes used as references.

Once imported by means of specific software tools, each 3D-volumetric data set was standardized using reference planes. The three planes are shown in Figure 1 and are defined by an axial plane passing through right and left infraorbitale points as well as right porion; a coronal plane passing through left and right porion perpendicular to the axial plane of choice; and a sagittal plane passing through the nasion point perpendicular to the axial and coronal planes of choice.

**Figure 2:**
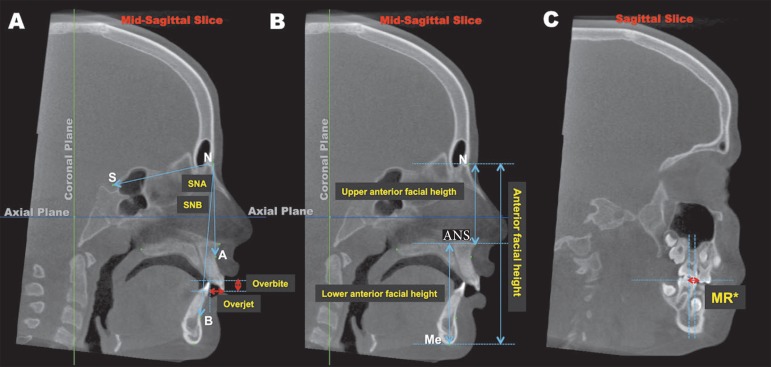
Sagittal slice with landmarks and measurements. A) SNA, SNB, overbite and overjet; B) N-Me, N-ANS and ANS-Me; C. MR* (right molar relationship and left molar relationship).

After standardization of head positioning, anatomical points (Sella, Nasion, A point, B point, Anterior Nasal Spine, and Menton) were analyzed through mid-sagittal slice images. Subsequently, landmarks 0.025 mm in diameter were identified (Table 1). The following measurements were performed (Fig 2): SNA (anteroposterior maxillary position), SNB (anteroposterior mandibular position), ANB (anteroposterior maxillo-mandibular relationship), N-ANS (upper anterior facial height), ANS-Me (lower anterior facial height), N-Me (anterior facial height), overjet, overbite, rMR (right molar relationship), and lMR (left molar relationship). Molar relationship was determined as the perpendicular distance from the tip of mesiobuccal cusps of upper first permanent molar to the mesiobuccal sulcus of lower first permanent molar on the same side. Values of rMR and lMR could not be obtained at T_2_ because of the artefacts caused by orthodontic bands in these CBCT images.

**Table 1:** Definition of landmarks

Landmarks (abbreviation)	Definition
Orbitale (Or)*	Most inferior point on infraorbital rim
Porion (Po)*	Most superior point of anatomic external auditory meatus
Nasion (N)	Midsagittal point at junction of frontal and nasal bones at nasofrontal suture
Sella (S)	Midpoint of rim between anterior process at mid-sagittal plane
A point (A)	Deepest point of the maxillary alveolar bone concavity at mid-sagittal plane
B point (B)	Deepest point of the mandibular alveolar concavity at mid-sagittal plane
Anterior nasal spine (ANS)	Most anterior limit of floor of nose, at tip of ANS at mid-sagittal plane
Posterior nasal spine (PNS)	Most posterior point along palate at mid-sagittal plane
Menton (Me)	Most inferior point along curvature of chin at mid-sagittal plane

* Bilateral landmark

Measurements were performed separately at each time (T_1_, T_2_ and T_3_) by the same examiner with a one-week interval in between. Intraexaminer reliability values were determined by means of intraclass correlation coefficient (ICC), with 95% confidence interval. Fifteen CBCT scans were randomly selected and remeasured by the same examiner (CB) within 2 weeks, under the same conditions, and compared with the first measurements. All measurement error coefficients were found to be close to 1.00 and within acceptable limits (higher than 0.95, except for MR measurement that was 0.91). The mean measurement difference obtained was less than 0.4 mm and 0.3º, which was considered not significant.

Means, standard deviations, minimum and maximum values were calculated for each measurement. After finding normal data distribution by means of the Kolmogorov-Smirnov non-parametric test, statistically significant differences were identified using paired Student's t-test (P < 0.05 - 95% interval confidence) between T_2_ and T_1_, T_3_ and T_2_, and T_3_ and T_1_. The percentage of patients who had the same qualitative mean changes during the interval T_1_-T_3_ was also calculated. Patients were considered to have increased measurement (mean difference ≥ 0.5 mm); no change (-0.5 mm >and <0.5 mm); and decreased measurement (≤ -0.5 mm). Statistical analysis was carried out using the SPSS software version 16.0 (SPSS Inc., Chicago, IL, USA).

## RESULTS

Separation of the mid-palatal suture was clinically confirmed in all patients with increased opening of inter-incisor diastema or within 3-5 days following expander activation. These data were confirmed on the CBCT image at T_2_. During the retention period, one of the patients returned without the expander, thus, his data were not computed at T_3_. Transverse deficiency was corrected in all patients. Data of RME transverse effects were previously published.^10^

Table 2 shows the descriptive analysis (minimum, maximum and standard deviation) of measurements obtained before treatment onset (T_1_), immediately after expansion (T_2_) and after retention (T_3_). Table 3 shows Student's t-test results yielded between the following intervals: T_2_-T_1_, T_3_-T_2_ and T_3_-T_1_. Significant differences were identified immediately after RME (T_2_-T_1_) as the maxilla moved forward (SNA mean increase was 1.21º), the mandible moved downward (ANS-Me mean increase was 0.97 mm and N-Me mean increase was 1.44 mm), overjet increased in 1.4 mm and overbite decreased in 1.76 mm. During the retention period (T_3_-T_2_), the maxilla relapsed backward (SNA mean decrease was 0.83º) and the mandible was displaced forward (SNB mean increase was 0.78º), improving Class II ANB relationship (mean decrease of 1.61º), although patients were left with an overall increase in anterior facial height.

**Table 2:** Descriptive analysis of measurements obtained in before treatment onset (T_1_), immediately after expansion (T_2_) and after retention (T_3_).

	T_1_ (n = 17)			T_2_ (n = 17)			T_3_ (n = 16)		
	Min.	Max.	Mean ± SD	Min.	Max.	Mean ± SD	Min.	Max.	Mean ± SD
SNA	74.38	86.20	79.71 ± 3.31	76.88	86.07	80.92 ± 2.99	76.53	86.07	80.09 ± 2.98
SNB	66.8	77.2	73.15 ± 3.41	67.71	76.6	72.92 ± 2.66	69.34	77.6	73.7 ± 2.72
ANB	4.00	9.49	6.61 ± 2.10	4.24	10.70	8.00 ± 2.25	2.50	10.03	6.39 ± 2.03
N-ENA	36.94	55.80	46.87 ± 4.54	36.84	56.52	47.27 ± 5.34	37.93	56.66	47.92 ± 4.76
ENA-Me	53.96	71.87	60.33 ± 4.16	56.59	74.23	61.30 ± 4.31	54.92	73.97	60.75 ± 4.41
N-Me	95.21	116.7	107.2 ± 6.06	94.96	117.57	108.6 ± 6.66	96.18	119.22	108.7 ± 6.51
*Overjet*	3.5	13.7	7.98 ± 3.56	3.51	14.67	9.38 ± 3.49	3.94	12.4	7.5 ± 2.78
*Overbite*	1.35	6.68	4.36 ± 1.61	0	5.5	2.59 ± 1.79	1.62	7.67	4.51 ± 1.78
RMd	0.5	9.09	3.18 ± 2.5	---	---	---	-2.68	6.83	1.84 ± 2.76
RMe	0.5	8.33	3.56 ± 2.27	---	---	---	-2.83	7.7	2.04 ± 2.59

n = number of patients; Min = minimum; Max = maximum; SD = standard deviation.

**Table 3:** Results regarding skeletal and dental changes between pre-treatment and post-expansion (T_2_ - T_1_), post-retention and post-expansion (T_3_ - T_2_), and post-retention and initial (T_3_ - T_1_).

	T_2_-T_1_ (n = 17)	T_3_-T_2_ (n = 16)	T_3_-T_1_ (n = 16)
	Mean ± SD	Mean ± SD	Mean ± SD
SNA	1.21* ± 1.96	-0.83* ± 1.28	0.38 ± 1.32
SNB	-0.23 ± 2.05	0.78* ± 1.26	0.55 ± 1.76
ANB	1.39*** ± 1.09	-1.61*** ± 1.32	0.22 ± 0.84
N-ENA	0.40 ± 1.88	0.65 ± 1.31	1.06* ± 1.45
ENA-Me	0.97* ± 1.40	-0.55* ± 0.90	0.42 ± 1.40
N-Me	1.44*** ± 1.82	0.02 ± 1.18	1.46*** ± 1.42
*Overjet*	1.4* ± 1.96	-1.87*** ± 1.50	-0.47 ± 1.33
*Overbite*	-1.76*** ± 0.72	1.91*** ± 0.92	0.15 ± 0.56
RMd	---	---	-1.33** ± 1.23
RMe	---	---	-1.55** ± 1.55

n=number of patients; SD= Standard Deviation; Level of significance =

* P < 0.05

** P < 0.01

*** P < 0.001.

Table 4 shows a qualitative description of changes found within T_1_-T_3_. Class II dental relationship (rMR and lMR) improved in 75% of patients.

**Table 4:** Number and percentage of patients with increased (≥ 0.5), no changes (- 0.5 > and < 0.5) or decreased (≤ - 0.5) measurements during the interval T_1_-T_3_.

T_1_-T_3_			
	Increased n(%)	No changes n(%)	Decreased n(%)
SNA	6 (37.5)	9 (56.25)	1 (6.25)
SNB	8 (50)	5 (31.25)	3 (18.75)
ANB	5 (31.25)	3 (18.75)	8 (50)
N-ENA	13 (81.25)	3 (18.75)	----
ENA-Me	8 (50)	5 (31.25)	3 (18.75)
N-Me	13 (81.25)	2 (12.50)	1 (6.25)
Overjet	4 (25)	2 (12.5)	10 (62.5)
Overbite	3 (18.75)	11 (68.75)	1 (6.25)
RMd	1 (6.25)	3 (18.75)	12 (75)
RMe	1 (6.25)	3 (18.75)	12 (75)

n = number of patients.

## DISCUSSION

This study is part of a long-term prospective clinical investigation into the effects of RME on Class II malocclusions using CBCT imaging.^10,11^ Understanding the effects of RME on Class II, Division 1 patients is of paramount importance, since transverse maxillary deficiency is often associated with this malocclusion.

Immediately after RME therapy, Class II relationship was worse in the anteroposterior and vertical dimensions. The maxilla significantly moved forward, whereas the mandible moved backward to a lesser degree. Skeletal changes were previously reported by Haas^12^ and have been recently confirmed by meta-analysis^13^ and systematic reviews.^14-16^ Dental changes mirrored skeletal changes by showing significant increase in overjet and decrease in overbite. Changes in dental and skeletal relationships were more likely to be associated with premature contacts involving palatal cusps and dental-alveolar inclination caused by RME^17^ than to inferior displacement of the maxilla. This effect was confirmed by the significant increase in buccal inclination (7.31°/6.46°)^10^ found in upper first molars immediately after RME.

The 6-month retention period with the Haas expander did not only maintain the new skeletal, alveolar and dental transverse dimensions, (1.66 mm, 4.69 mm and 5.89 mm, respectively, P < 0.001),^10^ but also resulted in significant decrease in dentoalveolar angulation of original levels. The wider maxilla allowed mandible to shift forward more than upward, therefore improving skeletal and dental relationships. This was revealed by overjet decrease, overbite increase and MR improvement.

By the end of the assessment period, sagittal skeletal changes were not significantly different when compared with initial data, except for patient's vertical dimension. However, Class II dental relationship significantly improved in 75% of patients. Studies assessing untreated Class II malocclusions determined that dental and skeletal patterns were not self-corrected,^3,18^ but became even worse.^19^ Wendling et al^20^ observed that some patients had spontaneous Class II correction after RME during the retention period (6-12 months) in cases of moderate Class II malocclusions. McNamara et al^21^ recently observed great improvement (1.8 mm) in MR after RME therapy in 81% of Class II patients when compared to non-treated controls (0.3 mm).

No statistically significant vertical changes were identified immediately after RME. This differs from previous studies that used cephalometric imaging^5,22-25^ and reported downward displacement of the maxilla. However, after the retention period, a significant increase in the superior anterior facial height was observed in 81.25% of patients examined herein (N-ANS increased 1.06 mm). In contrast to RME active phase, the retention period was longer which could possibly explain the vertical growth of the maxilla over this period.^26,27^ It is expected that untreated 9-year-old subjects would undergo vertical growth of 1.5 mm per year for boys and 1.2 mm for girls.^26^ Mc Namara et al^21^ observed a facial height increase of 3.4 mm in a RME group and 4.2 mm in the control group over a mean observation period of 3.7 years.

Despite the fact that the present study only assessed Class II Division 1 malocclusion patients, the severity of malocclusion was not considered (Table 2). The large variability of skeletal involvement may precipitate different responses to the same therapy. Vertical changes, resulting either from RME or growth, may limit horizontal mandibular changes and hinder forward positioning of the menton.^28^ Vertical maxillary control during the active phase and the retention period would allow further anterior repositioning of the mandible.

The number of patients included in the present study, although sufficient to detect statistically significant changes, is likely insufficient to generalize the results to all Class II malocclusions. The lack of a control group was a limitation of the present study; however, a control group was unfeasible for the present study due to ethical reasons, since it is impossible not to intervene when a diagnosed transverse discrepancy is present.

The routine use of CBCT is not recommended for orthodontic procedures, given that conventional images emit lower radiation doses. However, some orthodontic patients require temporomandibular images, frontal and lateral cephalograms, panoramic, periapical, occlusal or bite-wing radiographs. It is worth noting that the effective dose related to a full-mouth radiographic survey, as reported by Gibbs,^8^ and the sum of the effective doses for panoramic, lateral cephalometric and periapical images are similar, if not higher than that of CBCT without a 3D evaluation. This study used CBCT images because a 3D evaluation had also been carried out for other analyses and some data had already been previously reported.^10,11^

## CONCLUSIONS

A 6-month retention period with the Haas expander after RME therapy in Class II Division 1 malocclusion patients allowed the mandible to be positioned significantly more forward and exhibit an improved anterior position rather than an inferior position. This improved Class II dental relationship in 75% of the patients evaluated.
